# A digitally driven manufacturing process for high resolution patterning of cell formations

**DOI:** 10.1007/s10544-023-00655-1

**Published:** 2023-04-21

**Authors:** Matthew A A Smith, M Ibrahim Khot, Silvia Taccola, Nicholas R Fry, Pirkko L Muhonen, Joanne L Tipper, David G Jayne, Robert W Kay, Russell A Harris

**Affiliations:** 1 Faculty of Engineering and Physical Sciences, University of Leeds, Leeds, LS2 9JT UK; 2 Faculty of Medicine and Health, University of Leeds, Leeds, LS2 9JT UK; 3 Faculty of Biological Sciences, University of Leeds, Leeds, LS2 9JT UK; 4 School of Biomedical Engineering, University of Technology Sydney, Ultimo, NSW 2007 Australia

**Keywords:** Aerosol jet printing, Digital manufacturing, Microscale patterns, Cell patterning, *In vitro* cell models

## Abstract

This paper presents the engineering and validation of an enabling technology that facilitates new capabilities in *in vitro* cell models for high-throughput screening and tissue engineering applications. This is conducted through a computerized system that allows the design and deposition of high-fidelity microscale patterned coatings that selectively alter the chemical and topographical properties of cell culturing surfaces. Significantly, compared to alternative methods for microscale surface patterning, this is a digitally controlled and automated process thereby allowing scientists to rapidly create and explore an almost infinite range of cell culture patterns. This new capability is experimentally validated across six different cell lines demonstrating how the precise microscale deposition of these patterned coatings can influence spatiotemporal growth and movement of endothelial, fibroblast, neuronal and macrophage cells. To further demonstrate this platform, more complex patterns are then created and shown to guide the behavioral response of colorectal carcinoma cells.

**Graphical Abstract:**

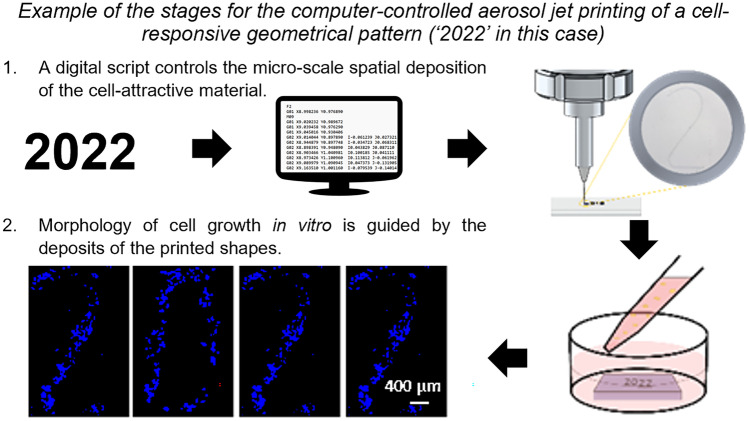

**Supplementary Information:**

The online version contains supplementary material available at 10.1007/s10544-023-00655-1.

## Introduction

*In vitro* cell models are used in pre-clinical evaluations (Stock et al. 2016; Jia et al. 2017) and tissue engineering (Espinosa-Hoyos et al. 2018). However, the gap between simple *in vitro* models and *in vivo* models can often result in the poor translation of findings, leading to high attrition rates in drug discovery and uncertainty of clinical relevance (Waring et al. 2015). Therefore, more complex and tailored *in vitro* cell environments need be created, which better mimic *in vivo* conditions and improve the quality of laboratory-based investigations (Cersosimo et al. 2014; Nikolić et al. 2017).

*In vitro* tissue architecture can be modelled by guiding the growth of cells by the selective deposition of microscale topographical and chemical cues on homogeneous 2D cell culturing platforms (Teixeira et al. 2003; Mirbagheri et al. 2019; Zhang et al. 2019; Shi et al. 2020). These highly structured surfaces composed of various patterns, also known as 2½ dimensional objects (Mirbagheri et al. 2019), have previously been shown to influence the tethering, movement, proliferation and differentiation of cells, with cell morphological and functional responses greatly depending on the cell type as well as the pattern type and dimensions (Hynes 2009; Khan et al. 2010; Jeon et al. 2015; De Silva et al. 2006). To date, a significant challenge in realising material surfaces with complex chemistries and topographies is the availability of effective and efficient manufacturing processes (Shi et al. 2020). The majority of the methods require multiple steps and combinational approaches that include template-based methods such as photolithography, microcontact printing and soft lithography (Kleinfeld et al. 1988; Hughes et al. 2014; Valentini et al. 1994; Welle et al. 2005; Hirschbiel et al. 2015; James et al. 2000; Bing et al. 2010; Bernard et al. 2000; Nie and Kumacheva 2008). Although these methods have been established in the laboratory, they are accompanied by significant lead-times and costs which do not effectively support mass customisation and are not amenable to adaptation and iterative development of novel material formulations due to their template-based nature. To overcome these limitations, easy-to-access and straightforward approaches need to be explored. Direct-write techniques such as inkjet (Murphy and Atala 2014) and extrusion-based printing (Ning and Chen 2017), have used the direct deposition of live cells which can cause high mortality rates due to excessive forces on the cells and are often limited by material selection and offer limited printing resolution, with a minimum feature size of ~50 μm (Murphy and Atala 2014). Having the manufacturing capability to rapidly screen a wider range of biomaterials at a smaller scale with high design flexibility, would not only facilitate the concept of rapid prototyping to biological assays, but it also paves the way for personalised *in vitro* models.

In this work a digitally-driven aerosol jet manufacturing process is presented that enables highly complex patterns to be created, while being flexible and responsive to changing design needs. This approach is enabled by the creation of a bespoke manufacturing apparatus and methodology that provides pattern fabrication with micron scale resolution (~10 µm) through Aerosol Jet Printing (AJP) (Secor 2018; Wilkinson et al. 2019). AJP is based on the focused deposition of an aerosolized material and, by actuating the print head and flow interruption, this machine can combine microscale features and thin films to create customizable patterns (Fig. S1). Significantly for microscale deposition, AJP can be used to pattern onto both planar and non-planar substrates owing to its large nozzle stand-off distance (~5 mm). Unlike other methods, AJP allows an extensive range of materials to be deposited, dependent on their ability to transition from the liquid/suspension state to an aerosol state (Strale et al. 2016). Previously AJP has primarily been explored in electronics manufacturing applications, but it has also been used to print polymers (Hegge et al. 2011; Zare Bidoky and Frisbie 2016), metal nanoparticles (Tamari et al. 2014; Maiwald et al. 2010; Rahman et al. 2016), ceramics (Große Holthaus and Rezwan 2008), and proteins (Grunwald et al. 2010) onto a wide range of substrate materials and surface finishes (Marinov et al. 2007; Schuetz et al. 2014; Numan-Al-Mobin et al. 2014; Kell et al. 2017; Rahman et al. 2015; Zhao et al. 2012; Wang et al. 2016). In this paper, computer controlled AJP deposition enables the creation of cell-responsive patterns using a two-step process (De Silva et al. 2006) to manufacture the extracellular environment and then introduce cells to this, forming a functionalized model. The proposed technology is demonstrated by manufacturing cell patterning environments comprised of microscale poly(3,4-ethylenedioxythiophene) polystyrene sulfonate (PEDOT:PSS) features on the surface of poly(dimethylsiloxane) (PDMS) substrates; a combination which provides a respective attractive/non-attractive cell adhesion response. When cells were cultured onto these substrates the surface treatment invoked a controllable response in cell adhesion and subsequent directionality of the cell growth according to the printed patterns. The effect was shown in six cell lines selected to represent significant varieties of cell types and to illustrate the new options of cell modelling in important clinical study areas. We believe that the technology described in the paper represents a major advancement as it permits the creation of complex cell environments with the scales and substrate materials widely used in lab-on-a-chip devices, whilst newly enabling greater speed, flexibility, ease of use, and control for the users. It will support new developments in lab-on-a-chip devices across a wide range of studies and techniques and consequently enable new discoveries and applications.

## Materials and methods

### Material feedstock formulation

A solution containing PEDOT:PSS (Clevios PH1000TM, Heraeus Holding) was diluted with Ethylene Glycol (20% v/v, > 99.8%, Sigma Aldrich) and 3-glycidyloxypropyl)trimethoxysilane (GOPS) (0.2% v/v, > 98%, Sigma Aldrich) and used as the print material (Capel et al. 2021). PEDOT:PSS particles are suspended in the solvents, so the suspension was ultrasonically agitated for 10 min prior to printing to break up agglomerates and disperse the particles.

### Substrate preparation

PDMS substrates were cast into a glass petri dish to a thickness of a few millimetres, cured at T = 100 °C for 35 min in an oven, and cut in the desired dimension (10 mm × 10 mm). Then, they were ultrasonically washed in acetone for 5 min, followed by a further wash in isopropanol. The substrates were then rinsed with type 2 deionized water and dried under a nitrogen gas stream. The substrates were treated for 1 min with oxygen plasma immediately prior to printing to create a hydrophilic surface and facilitate the spreading of the PEDOT:PSS water dispersion and the printing of homogeneous patterns.

### Aerosol jet printing and post processing

The prepared PEDOT:PSS formulation was processed in the ultrasonic atomizer of the aerosol jet printer (Optomec Aerosol Jet print engine, Optomec Inc.). Nitrogen was used as the inert sheath and atomiser gas. A 100 µm nozzle, sheath gas flow rate of 40 SCCM, carrier gas flow rate of 13 SCCM, stage speed of 1.7 mm/s and Z height above the substrate surface of 2.5 mm were used throughout. Gas flow rates are quoted in standard cubic centimetres per minute (SCCM). After printing, the substrates were placed in an oven at 100 °C for 10 min to drive off solvents and make the PEDOT:PSS resistant to water.

### Analysis of deposits

Geometrical data was obtained from a Zygo NewView 5000 white light interferometer. Cross sectional data was analyzed for the maximum height, width at half maximum height, width at the base and cross-sectional area.

### Cell culture procedure

The printed PDMS substrates were sterilized in ethanol (70% v/v) for 10 min, prior to cell culturing. Mouse fibroblast L929 (ATC CCL^−1^), mouse macrophage RAW264.7 (ATCC TIB-71), the human endothelial cell line EA.hy926 and the colon cancer HT-29 cell lines were maintained in Dulbecco’s modified Eagle’s medium DMEM supplemented with Fetal Bovine Serum (10% v/v, FBS), L-Glutamine (2 mM), penicillin (100 I.U./ml) and streptomycin (100 µg‧ml^−1^). BHK cells (Invitrogen R700-01) were maintained in Glasgow MEM and C6 (ATCC CCL107) cells in Hams F12 medium supplemented with L-Glutamine (2 mM), FBS (10% v/v), penicillin (100 I.U./ml) and streptomycin (100 µg‧ml^−1^). The colorectal cancer cell line, HCT-116, was obtained from the European Collection of Authenticated Cell Cultures (ECACC, Salisbury, England). Cells were cultured in RPMI 1640 + GlutaMAX cell culture medium (Gibco^®^ by Life TechnologiesTM, Paisley, UK) supplemented with FBS (10% v/v), penicillin (100 units‧ml^−1^) and streptomycin (100 μg‧ml^−1^).

Cell cultures were maintained at 37 °C in CO_2_ (5% v/v), with 95% (v/v) relative humidity. Upon reaching 90% confluency, cell cultures were briefly washed with Dulbecco’s phosphate-buffered saline (DPBS) and incubated with trypsin (0.05% v/v) and ethylenediaminetetraacetic (0.5% v/v) acid for 5 min. Medium containing FBS (10% v/v) was then added to trypsinized cells in suspension, which were then centrifuged at 400 g for 5 min and resuspended in fresh cell culture medium. Cells were seeded at 7.5 × 10^4^ cells per PDMS substrate and incubated for the specified time period before being imaged using an EVOS FL Cell Imaging System (Thermo Fisher Scientific, Altrincham, UK).

### MTT assay

Samples were placed in three wells of a 6-well plate. The other three wells were used as a control. Cells were seeded at 7.5 × 10^4^ cells per PDMS substrate and incubated for 24, 48 and 72 h. MTT colorimetric assay was performed to measure cell viability at the end of each incubation period. MTT dye was dissolved at a final concentration of 1 mg‧ml^−1^ in cell culture medium. The medium was removed from wells and 3 ml of MTT solution was added to cells and incubated for 3 h. After 3 h, the media was carefully removed from the wells and MTT-formazan crystals were dissolved by the addition of 2.5 ml of isopropanol. The absorbance was measured on 100 μl of solution in a 96 wells plate at 570 nm wavelength by using a Berthold Technologies microplate reader. The experiment was repeated three times.

### Live/dead assay

Samples were placed in the wells of a 6-well plate. Cells were seeded at 7.5 × 10^4^ cells per PDMS substrate and incubated for 24, 48 and 72 h. Live/Dead assay was performed to measure cell viability at the end of each incubation period. 10 μl of propidium iodide and 10 μl of Hoechst 33342 were dissolved in 80 μl of PBS. This solution was added to the well and incubated for 30 min. After 30 min, fluorescent imaging was performed on a EVOS FL microscope. Red fluorescence generated by propidium iodide stain was used to indicate dead cells and the blue fluorescence of Hoechst 33342 stain demonstrated both live and dead cells.

### SEM sample preparation and imaging

Cells cultures were washed with pre-warmed (DPBS) and fixed in glutaraldehyde (2.5% v/v) for 20 min. Cells were then subjected to dehydration in increasing gradients of ethanol (25%, 40%, 60%, 80%, 90% and 100% v/v). For imaging, samples were coated with 4 nm of iridium and imaged using a FEI Nova NanoSEM (Thermo Fisher Scientific).

## Results and discussion

**Fig. 1:**
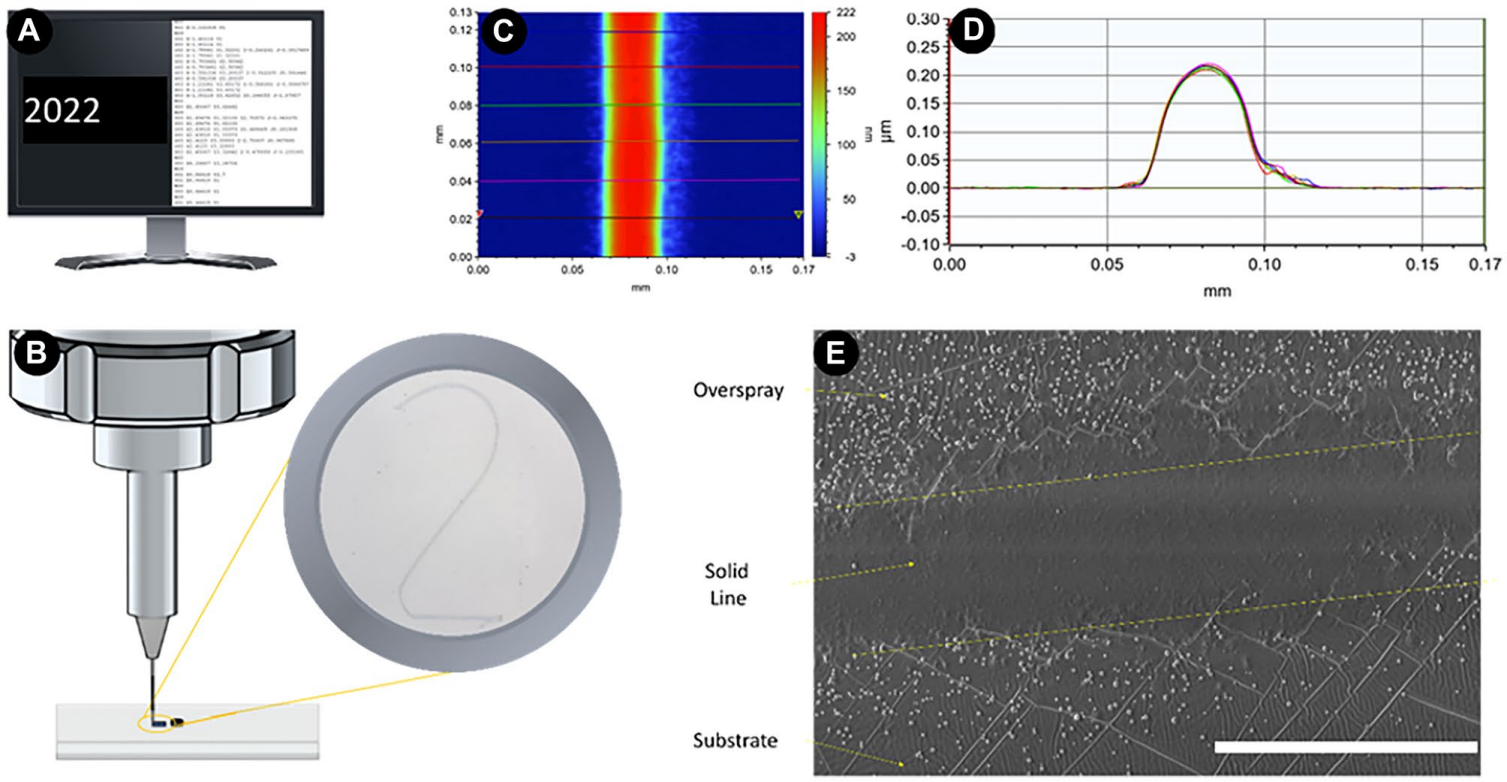
**A** Program containing manipulation instructions is generated from digital design data **B** Acellular material is deposited directly onto the substrate surface. **C** Representative white light interferometry measurements of a PEDOT:PSS linear track on a PDMS substrate used to assess the morphology of the printed lines (Red = Peak, Blue = Substrate surface). **D** Multiple (n = 6) two point profiles confirming the consistency of the line cross sectional geometry. **E** SEM analysis of a line showing the overspray to the sides of the printed features. Scale Bar = 40 µm

The AJP system used in this study comprises of a high resolution 5-axis stage which moves the substrate below the aerosol stream under Computer Numerically Controlled (CNC) actuation. The print design is first created in standard graphics or Computer Aided Design (CAD) software before being translated to machine control code (G-Code), Fig. 1A. The material feedstock is then aerosolized, transported, and deposited as a focused beam onto the substrate, Fig. 1B. Because there is a continuous flow of aerosol, a high-speed mechanical shutter with a response time of 2 ms is used to interrupt the flow of material where discrete deposits are required (Fig. S1). The linear translation stages give a minimum incremental movement of 100 nm and a 300 mm travel distance with a maximum speed of 400 mm/s in the XY plane. Combining this with a range of focusing nozzle apertures, micro to macro scale patterning is possible. In addition to the size of the nozzle, the geometry of the printed deposit is affected by the speed the substrate is moved, and the two gas flow rates used to propel and focus the aerosol respectively.

Previously it was found that the geometry of linear tracks is predominantly affected by these gas flow rates (Smith et al. 2018, Capel et al. 2021), confirming results by Mahajan et al*.* (2013). These parameters can be optimized to print well-defined lines with consistent, inverted parabolic shape and controllable dimension (i.e. maximum height, width at half maximum height, width at base) (Fig. 1C, D). As shown in other AJP work, these featured localized regions of particles along the edges which are termed overspray (Chen et al. 2018) (Fig. 1E).

In other published work, we have demonstrated the use of AJP to reliably produce micro scale cell attractive patterns in the region of 20 μm wide from poly(3,4-ethylene dioxythiophene):poly(styrene sulfonate) (PEDOT:PSS) onto both glass and poly(potassium 3-sulfopropyl methacrylate) (PKSPMA) coated glass substrates (Capel et al. 2021). PEDOT:PSS was used as the patterning material because of its unique set of properties, such as the excellent biocompatibility, the superior flexibility compared to inorganic conductors, the mixed ionic-electronic conductivity, which provides enhanced communication between cells and devices, and make it an amenable interface with biological tissues (Wang et al. 2016; Owens and Malliaras 2010; Higgins et al. 2012; Ohayon et al. 2017). In addition, PEDOT:PSS based substrates have been shown to allow the direct electrical stimulation of electrogenic cells (e.g. neurons and muscle cells), regulating or inducing several biological functions (Lundin et al. 2011; Gomez et al. 2007). Such electrical stimulation was not the subject of this work, but its use may permit such extensions in the future. These PEDOT:PSS micro features were shown to promote selective adhesion, growth and differentiation of SH-SY5Y neuroblastoma cells (Capel et al. 2021). In addition, we showed that the selective cell adhesion was enhanced by culturing SH-SY5Y cells onto PEDOT:PSS tracks deposited via AJP onto PKSPMA coated glass substrates, which inhibit cellular adhesion. This pairing of ‘attractive’ and ‘repulsive’ materials resulted in highly organised small-scale neural patterns, providing a new route to create bespoke neuronal culture environments (Capel et al. 2021). This prior successful use informed the material selection of PEDOT:PSS in this new study.

**Fig. 2:**
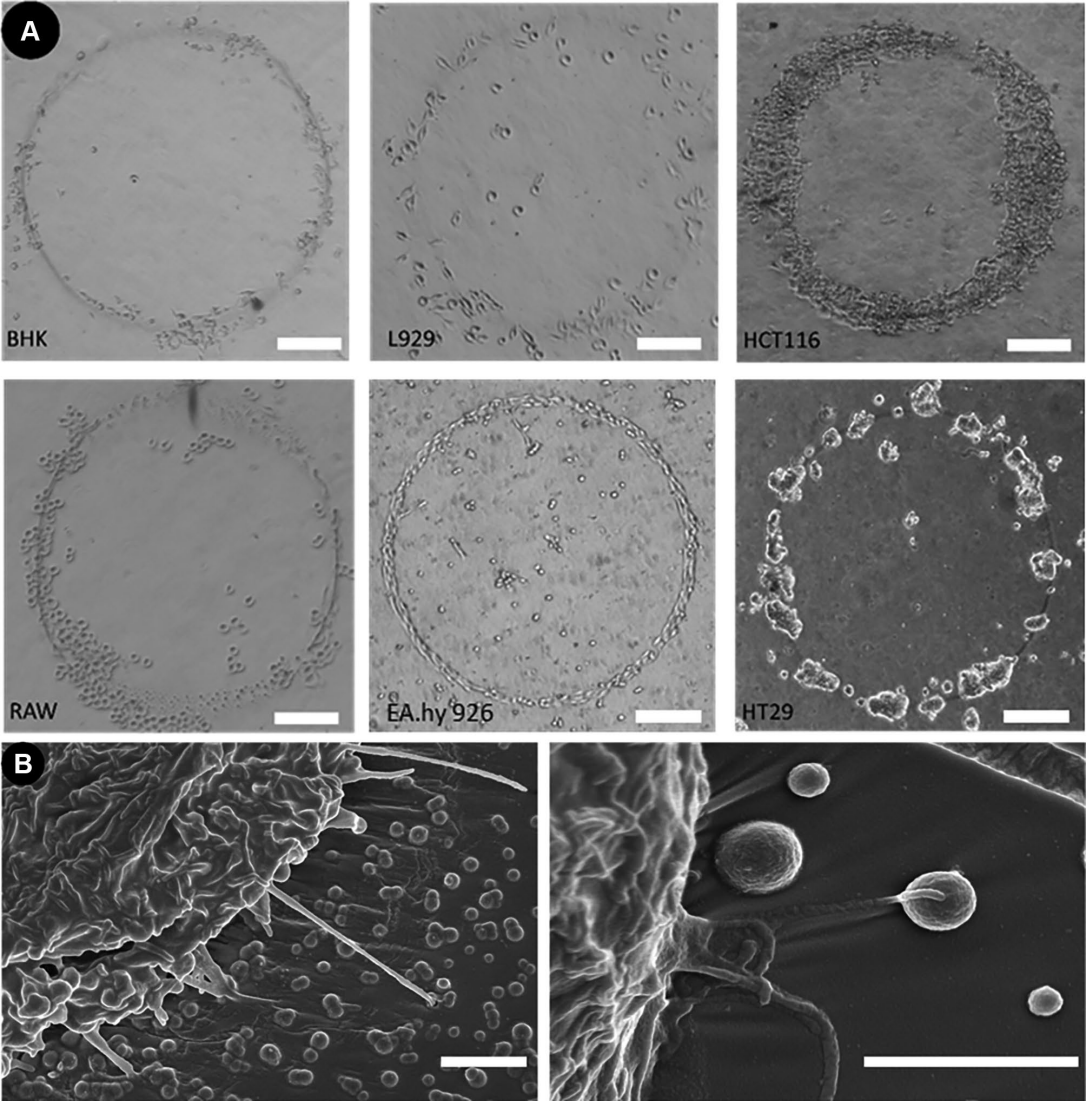
**A** A range of adherent cell lines all reacted to PEDOT:PSS printed lines on a PDMS substrate, and grew into circular shapes defined by the printed features. Scale Bars = 200 µm. **B** Immersion SEM images of HCT-116 cells (left of both images) extending “pseudopodia-like” projections to attach to the deposited particles of PEDOT:PSS. (Left) 10000X (Right) 25000X. Scale bars = 2 µm

In this present work, we have exploited this attractive/repulsive relationship but have done so with materials of greater interest that are widely used in lab-on-a-chip applications. Poly(dimethylsiloxane) (PDMS) substrates were used as it is biocompatible, optically transparent, facilitates the exchange of gases and the hydrophobic surface prevents cell adhesion (Van Midwoud et al. 2012) (Fig. S2). These properties make it an ideal candidate as the ‘repulsive’ substrate to complement our ‘attractive’ PEDOT:PSS printed patterns. PDMS was initially treated with oxygen plasma to create a hydrophilic surface and facilitate the spreading of the PEDOT:PSS water dispersion and the printing of homogeneous patterns. After the printing process, the surface undergoes ‘hydrophobic recovery’, returning to its natural state within a few hours (Hillborg et al. 2004). To avoid degradation/delamination of the printed patterns from PDMS substrates in aqueous environment, PEDOT:PSS dispersion was mixed with the silane based cross linking agent 3 glycidoxypropyltrimethoxysilane (GOPS) (Capel et al. 2021). The print parameters were set to print lines with a maximum height of 200 nm and a width at half maximum height of 30 µm (see Section 2 for details) (Fig. 1D). Once the extracellular environment has been manufactured, cells are introduced. Endothelial, fibroblast, neuronal, macrophage and carcinoma cell lines were selected to highlight the various uses of this concept in pre-clinical research. Cells were seeded directly onto the substrate surface and cultured over several days. Cell culturing treatment and analysis is described further in Section 2. As shown in Fig. 2A, the cells were found to preferentially adhere to the PEDOT:PSS deposits.

Morphology of cell growth was dependent on the cell type and was guided by the deposits into the printed shapes (1 mm diameter circles in Fig. 2 A and 5 mm long straight lines with 200 μm pitch in Fig. S3). Cells were found to favourably attach to the printed PEDOT:PSS, over the PDMS, through “pseudopodia-like” projections to both the printed lines and overspray (Figs. 2B and S4).

**Fig. 3:**
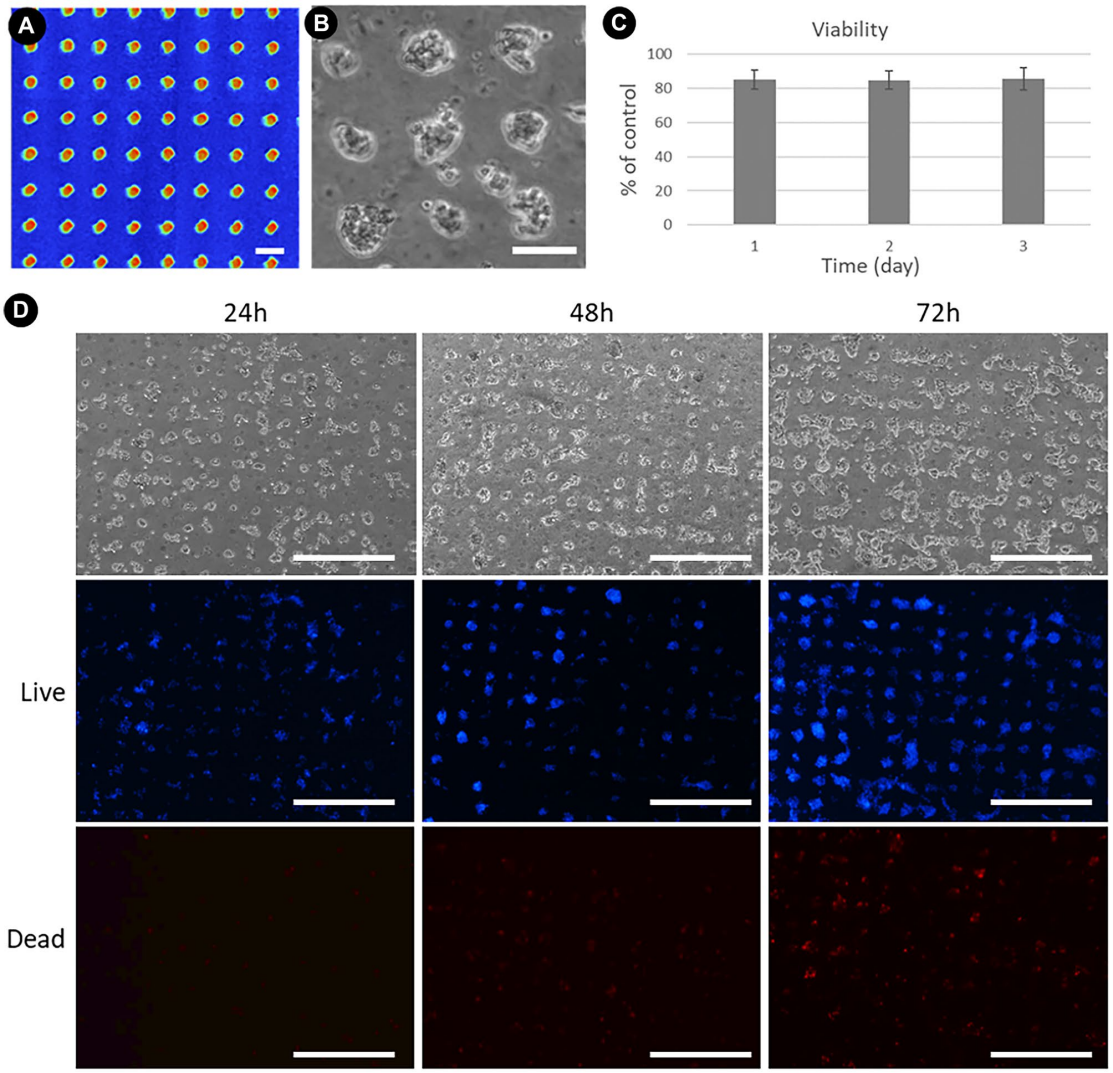
**A** White Light Interferometry traces of shaped design White light images; color indicates height, Red = Peak, Blue = Substrate; **B** HCT-116 cell response to an interrupted pattern, by growing on the printed features. Scale Bars = 100 µm. **C** Viability of cells assessed by MTT assay is more than 80% at three different time points (24, 48 and 72 h). **D** LIVE/DEAD fluorescent imaging cell staining of the HCT-116 cells incubated at different time points. Cells were incubated with Hoechst 33,342 (blue) and Propidium iodide (Red). Both live and dead cells are stained with blue fluorescence. Only dead cells are stained with red fluorescence. Scale Bars = 500 µm

Once the manufacturing process had been shown to influence cell growth, further studies were carried out using the HCT-116 colorectal carcinoma cell line, due to the potential value of this application in cancer research. A 20 × 20 matrix of 10 µm diameter at half height dots with a pitch of 110 µm was printed, to portray the ability of this apparatus in fabricating interrupted patterns (Fig. 3A).

Similar to the printed lines, cells were observed to preferentially attach to the PEDOT:PSS features. The HCT-116 cells were seen to create networks of localized cellular growth on the individual dots of printed material (Fig. 3B). Cell viability was assessed at 24, 48 and 72 h post cell seeding using the 3-(4,5-dimethylthiazol-2-yl)-2,5-diphenyl tetrazolium bromide (MTT) assay and LIVE/DEAD fluorescent imaging assay. As reported in Fig. 3C, MTT study showed at different time points no cytotoxic effect was observed to the HCT-116 cells on PDMS patterned substrates. Cell viability remained above 80%. LIVE/DEAD fluorescent imaging of HCT-116 cells incubated at different time points, confirmed no cytotoxicity in cells at all time points (Fig. 3D). Almost no dead cells can be observed on the surface of the sample, indicating the excellent biocompatibility of the proposed substrates. At 24 h, HCT-116 cells were observed to preferentially tether to the PEDOT:PSS dots and proliferate on the printed material. By 48 h, the dots were covered with cells. Once grown to confluency, cells created networks between the dots, which was typically observed between 48 and 72 h.

**Fig. 4:**
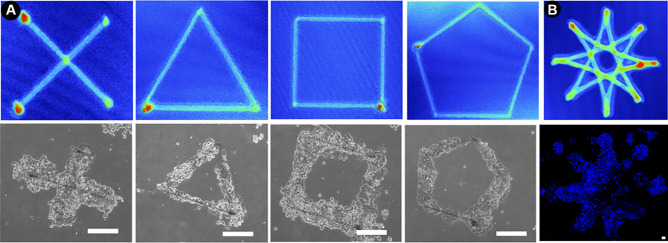
**A** White Light Interferometry traces of shaped designs and the response of HCT-116 cells to these prints after 48 h **B** The design can be scaled to pattern over larger areas. Scale Bars = 200 µm. White light images; color indicates height, Red = Peak, Blue = Substrate

Next, various shaped designs were created, to evaluate whether these can be used to formulate complex patterns. Cells were seen to align and grow to each pattern, suggesting the ability for fine control of cell cultures on small scales (Figs. 4A, S5, and S6). In addition to patterning over small areas, the printing parameters can be adjusted to influence cell growth on a larger scale (Fig. 4B). In this work, bespoke freeform patterns were selected to demonstrate the range of greater capability that this new technique newly enables. Ultimately, the specific application will dictate the cell lines and pattern required. Of course, further studies will be required for each specific application.

## Conclusions

In conclusion, we present a digitally-driven manufacturing process capable of microscale deposition of chemical and topographical features. A range of cell lines are shown to favourably attach and tether to these deposits, demonstrating the applicability of this process to many pre-clinical research areas. Our approach of first fabricating the extracellular environment means the technique is not limited to simply guiding cell growth. In the future, hybridization with other processes will allow the complexity of the model to be increased. Since the apparatus is flexible to allow the printing of a wide range of materials and to change designs on demand, it can enable the rapid testing and refinement of complex *in vitro* cellular models. Significantly, this process could also permit high-throughput, yet personalized, screening.

## Supplementary Information

Below is the link to the electronic supplementary material.

Supplementary file1 (PDF 547 KB)

Supplementary Information (SI) available:

- a schematic of the AJP technology (Fig. S1)
- images of representative controls (Fig. S2)
- images of a range of adherent cell lines align along straight lines pattern (Figs. S3 and S4)
- images of HCT-116 cells response to various sized and shaped patterns (Figs. S5 and S6)

## Data Availability

The data that supports the findings of this study are available from the corresponding author on request.
