# Supplementary material for: A digitally driven manufacturing process for high resolution patterning of cell formations
Source: Biomed Microdevices. 2023 Apr 21;25(2):16. doi: 10.1007/s10544-023-00655-1 (PMC10121500; doi:10.1007/s10544-023-00655-1)
Supplement: Supplementary file 1 — Supplementary file1 (PDF 547 KB) [file 10544_2023_655_MOESM1_ESM.pdf]

Supporting Information

A Digitally Driven Manufacturing Process for High Resolution Patterning of Cell Formations

*Matthew A A Smith, M Ibrahim Khot, S. Taccola, Nicholas R Fry, Pirkko L Muhonen, Joanne L Tipper, David G Jayne, Robert W Kay, and Russell A Harris**

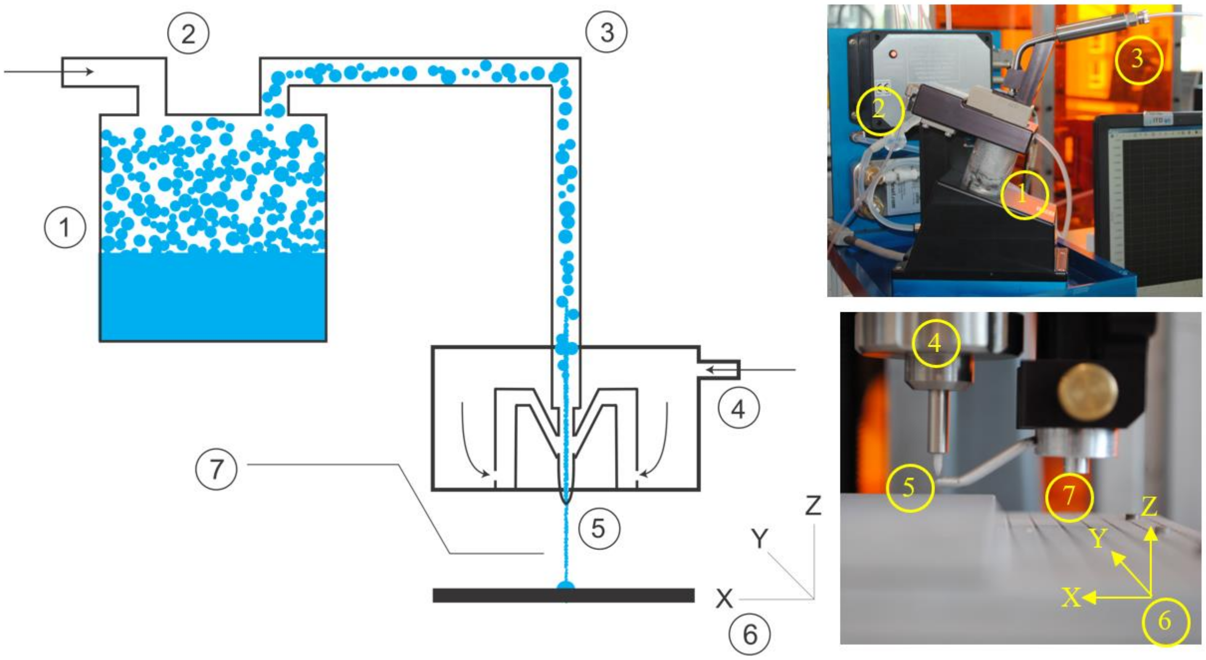

Figure S1: A liquid sample containing the functional material is atomized within a sealed container (1). Then, an inert gas is used to increase the pressure in the atomizer chamber and a mixed stream of nitrogen and aerosol is produced (2). This is transported to the deposition head, via flow refinement stages when appropriate, with the gas acting as a sheath around the aerosol (3). A further annular sheath of inert gas is introduced in the print head, which focuses and accelerates the aerosol (4) and the resulting high velocity jet is deposited onto the substrate (5). To produce a pattern the stage is moved in up to 5 axes (6), On/off patterning is achieved by interrupting the jet with a mechanical shutter (7) (Capel 2021).

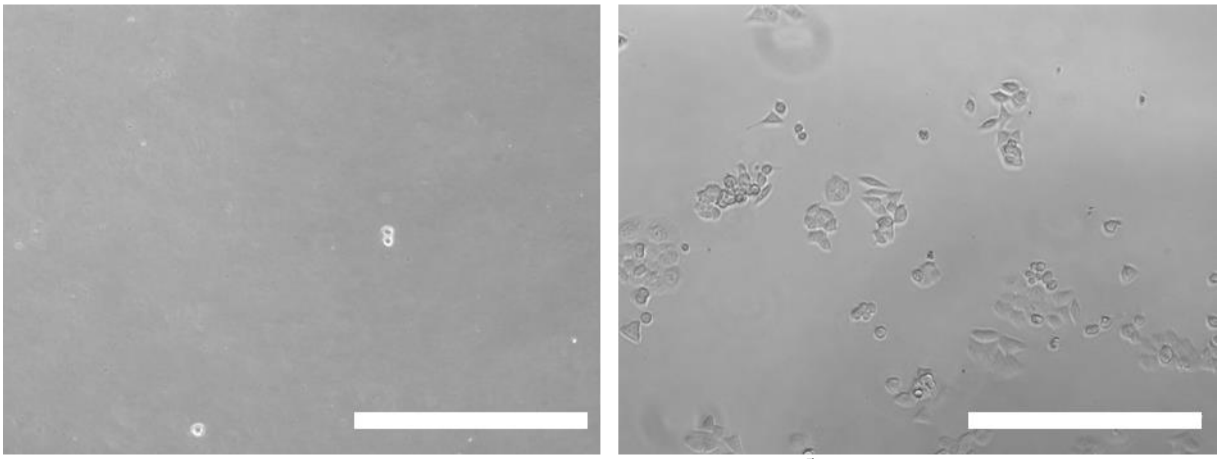

Figure S2: Representative controls seeded at the same cell density (5×10^5cells/ml) after 48 hours. (Left) PDMS Surface. (Right) Polystyrene Surface. Scale Bars = 400µm.

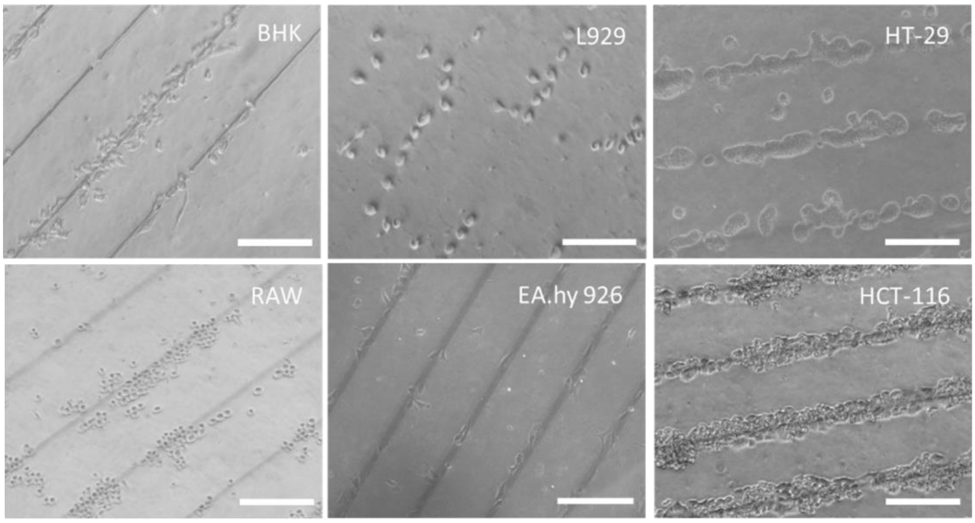

Figure S3: A range of adherent cell lines align along printed features. Scale Bars = 200 µm.

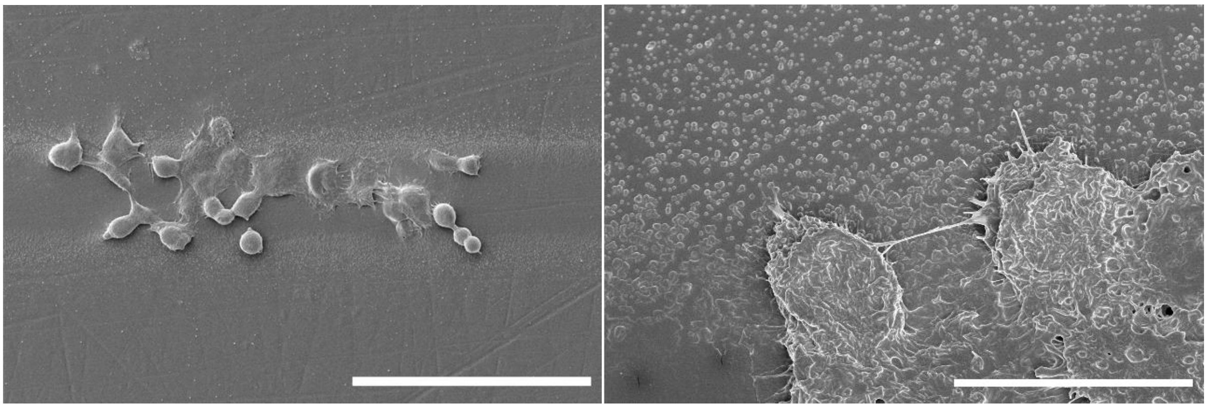

Figure S4: (Left) SEM image of HCT-116 cells tethering to PEDOT:PSS printed features. Scale Bar = 100µm (Right) HCT-116 cells extend projections to tether to the PEDOT:PSS prints over the PDMS substrate. Scale Bar = 20µm.

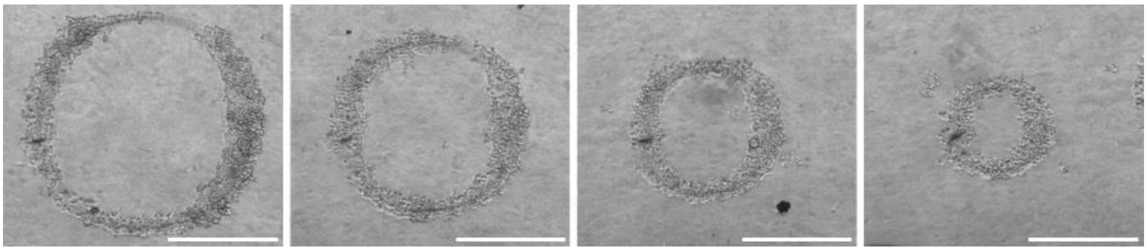

Figure S5: HCT-116 cell response to various sized PEDOT:PSS circles on PDMS substrates. Scale bars = 500µm.

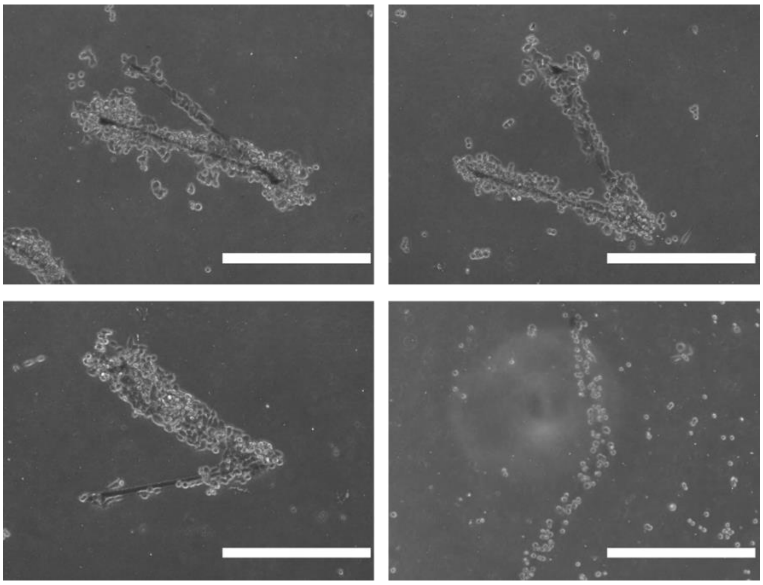

Figure S6: HCT-116 cells grow along corners of different angles after 24 hours. Scale bars = 400μm.
